# Supplementary material for: Dynamic modulation of pulsatile activities of oxytocin neurons in lactating wild-type mice
Source: PLoS One. 2023 May 10;18(5):e0285589. doi: 10.1371/journal.pone.0285589 (PMC10171594; doi:10.1371/journal.pone.0285589)
Supplement: Attachment — Submitted filename: Yaguchi et al Response to Reviewers.docx [file pone.0285589.s001.docx]

We greatly thank the editor and all the reviewers for their constructive suggestions and enthusiasm about our study. For the revised manuscript, we conducted four new experiments and two data analyses that, we believe, have addressed the reviewer’s comments and substantially improved our manuscript in terms of biological significance and the clarity of the text. Here, we summarize the major points and provide point-by-point responses to the reviewer’s comments. Line numbers correspond to those shown in the cleaned revised manuscript.

**Additional experiments:**

1) A new cohort of pup isolation experiments (Figure 3) to increase the sample size (Response #1-1).
2) A new cohort of pup fostering experiments to form new Figure 6 (Response #1-5).
3) Histochemical analysis of GCaMP6s expression in lactating females (Response #2-1)
4) Recording of OT neurons during pup retrieval (Response #2-2).

**Additional data analysis:**

1) Analyzing the peak height in Figure 4 (Response #1-3).
2) Analyzing the duration of the mother in the nest during the peri-weaning periods (Response #1-6).

**Point-by-point responses**

Reviewer #1:

In this interesting and compact study, Yaguchi et. al. used a newly generated AAV that express GcaMP6f in OT cells to record OT pulsatile activity during lactation. They demonstrated that OT pulsatile activity requires suckling in mice and revealed interesting dynamic changes in OT pulses over lactation days. The study is well performed and the tool will be useful for the neuroscience community. The manuscript is clearly written. There are only a few minor suggestions.

1. Figure 3C, error bar is missing. Please make sure that the data passes normality test before using t-test. This may not be the case given that the n is small, and the pulse number/magnitude of one animal is quite different from the rest three animals. For Number of pulses, it is not clear what the duration is. Please changes it to frequency of pulses or number of pulses/XX time.

Response #1-1:

We are greatly thankful to the reviewer for the highly favorable assessment of our main aims and conclusions. To address this important issue, we have obtained an additional cohort and collected a total of 7 data sets for the analysis through non-parametric statistics (Wilcoxon signed-rank sum test) to update Figure 3C. This analysis demonstrated that the intensity of photometric peaks remained unchanged, while their frequency showed a significant increase. As additional support, we present the cumulative distribution of IPIs in new Figure 3d. We have opted to exclude error bars in this and other figures, as all individual data points are visibly represented. The duration of these analyses was 2 hours, which we have explicitly indicated in the figure and accompanying legend.

2. Figure 4b, please indicate on the figure what gray and white shades mean.

Response #1-2:

We explicitly showed the meanings of gray and white shades in the legend of Figure 4b.

3. In Figure 4, is there any change in pulse magnitude across lactation days? From the representative trace, it looks like the pulse magnitude is higher during early lactation. Is that true across animals?

Response #1-3:

To address this important issue, we evaluated the peak height of the same animals during early (PPD2–4), middle (PPD12–14), and late (PPD18) lactation stages, and present our findings in new Figure 4c. Since we were unable to directly compare the precise values of ΔF/F across different animals, we calculated the fold change within each individual. As shown in Figure 4c, we have presented the fold change relative to the middle (PPD12–14) of the lactation stage. Two-sided one-way ANOVA did not reveal any statistical difference. Although our previous study demonstrated that the peak height increased from PPD1 to PPD12–14 (Yukinaga et al. Curr Biol 2022), no major change was observed between PPD2–4 and PPD12–14 in the current study. This suggests a substantial increase in peak height during the first one or two days of lactation. We added a short discussion on this point (lines 347–350).

4. Presumably, the same animal was recorded across days and light/dark cycle. All data should be paired. Please use lines to connect the dots that belong to the same animal. In such a way, it will be easier to see changes in behavior or pulse-related parameters across time. Two-way ANOVA (factor 1: light vs. dark; factor 2: PPD age) is more appropriate for analyzing Figure 4c-e.

Response #1-4:

We agree with this opinion and improved statistics and representations of new Figure 4e–g according to this suggestion.

5. As mentioned in the discussion, an interesting question is whether the change in IPI over lactation days is due to changes in OT system or changes in suckling behavior from the pups. As the pup age, presumably the suckling force will be different. This can be addressed by using older pups in early lactating females and younger pups in late lactating females. The authors are encouraged to address this question in the current study as it is quite related and straightforward.

Response #1-5:

We thank the reviewer for this encouragement. We have conducted an additional cohort to analyze the effects of fostering pups of different ages on the IPI distribution. As reported in our new Figure 6, we found that the age of pups could affect the clustering patterns of pulsatile activities. We have revised our original Discussion based on this finding.

6. The last figure was cut off at the bottom. Does the gradual decrease of OT pulses during peri-weaning stage reflect a gradual decrease of pup suckling? In other words, is the decrease in pulse frequency parallel to a decrease in female crouching time in the nest?

Response #1-6:

We sincerely apologize for the error in the initial submission and confirmed that we have corrected the issue in the revised manuscript. Our video analysis only provides information regarding the mother's presence in the nest (mostly touching with pups), but not the precise duration of suckling by the pups. To capture the latter, a high-magnification video taken from the bottom of the cage is required, which presents a significant challenge, even during the earlier lactation stages when the pups are relatively immobile. This approach has been successfully employed only for brief durations as reported in Yukinaga et al. Curr Biol 2022. Hence, our assessment of the time the mother spends in the nest (Figure 4) can only serve as a surrogate measure of mother–pup interaction. Despite this limitation, we analyzed the duration of the mother in the nest during the peri-weaning period, as shown in new Figure 7f, g. The mother's presence in the nest remained relatively stable across the PPD18–24 periods, which resulted in a reduction in the pulse numbers per 1 hour that the mother spent in the nest. Based on this data, it is difficult to provide a conclusive answer to the reviewer's question. To obtain a better understanding of the mother–juvenile interaction during the peri-weaning period, future studies incorporating automated video analysis will be required. We have clarified this limitation in the revised Discussion (lines 565–677).

Reviewer #2:

The authors described methods of photometric chronic measurements of calcium imaging by use of AAV vector that induces GCaMP6 expression under the control of a 2.6 kb mouse oxytocin mini-promoter. They found that pulsatile activities of oxytocin neurons require physical contact. The data are solid and very interesting.
Following points should be considered carefully.
1. Expression of GCaMP in oxytocin neurons was confirmed in non-pregnant females (C57BL/6N). This specific expression is also true in lactating animals and in ICR mice? At least discussion is necessary.

Response #2-1:

We are greatly thankful to this reviewer for providing a highly favorable assessment of our main aims and conclusions. To address this important point, we have added histological analysis to determine the specificity of GCaMP6s expression in lactating C57BL/6 mice (Figure 1b, c). Additionally, we have included a new discussion to explain that specific GCaMP6s expression is also attainable in ICR mice (lines 585–593).

2. The authors showed that oxytocin pulses occurred during the period when mothers were in the nest (quantitative data are preferable, if there are), suggesting that direct contact may be necessary for oxytocin pulses. However, is there any direct evidence for necessity of direct stimulation of “nipple”? Pulses occurred only during suckling not during just contact with pups? Are there no pulsatile activities during maternal behaviors of retrieval or licking?

Response #2-2:

This is a very interesting topic. In our previous study, we showed that simultaneous direct nipple stimulation by 3 pups was required to initiate pulsatile activities of OT neurons (Yukinaga et al. Curr Biol. Figure 2F). To further clarify this point, according to the suggestion of the reviewer, we analyzed the activities of OT neurons during pup retrieval and found no detectable pulsatile response (Figure 4d).

3. The authors showed that no oxytocin pulse was found under condition of indirect contact via mesh in the latter stage of lactation. Have you ever checked lactation mice that previously experienced lactation (mice of second or third lactation)?

Response #2-3:

We apologize that our explanation in the initial manuscript was not clear. For the pup isolation experiments, we utilized both primiparous and multiparous females (who nurse their second or third litters after weaning the previous ones). In the revised manuscript, we have augmented the sample size and provided explicit clarification regarding the maternal characteristics in the Method section (line 160–162) and the corresponding figure legend.
